# The Probiotic Potential and Metabolite Characterization of Bioprotective *Bacillus* and *Streptomyces* for Applications in Animal Production

**DOI:** 10.3390/ani14030388

**Published:** 2024-01-25

**Authors:** Alberto Gonçalves Evangelista, Tiago de Melo Nazareth, Carlos Luz, Victor Dopazo, Ana Moreno, Mario Riolo, Giuseppe Meca, Fernando Bittencourt Luciano

**Affiliations:** 1 Graduate Program in Animal Science, Pontifícia Universidade Católica do Paraná, Rua Imaculada Conceição, 1155 Prado Velho, Curitiba 80215-901, PR, Brazil; tiago@uv.es; 2 Departament Medicina Preventiva i Salut Pública, Ciències de l’Alimentació, Toxicologia i Medicina Legal, Facultad de Farmàcia, Universitat de València, Av. de Vicent Andrés Estellés s/n, 46100 València, Spain; carlos.luz@uv.es (C.L.); victor.dopazo@uv.es (V.D.); ana.moreno@uv.es (A.M.); mario.riolo@unirc.it (M.R.); giuseppe.meca@uv.es (G.M.)

**Keywords:** antimicrobial activity, *Bacillus*, *Streptomyces*, *Salmonella*

## Abstract

**Simple Summary:**

Given the imperative need to control *Salmonella* in animal production, and the escalating problem of antimicrobial resistance, there is a paramount requirement for alternative strategies that can uphold both animal productivity and health. In this context, the utilization of probiotics is an important option that needs to be further studied. This research encompassed a comprehensive analysis to assess the probiotic potential of *Bacillus* and *Streptomyces griseus*. The strains used exhibited significant inhibitory activity against *Salmonella* and the production of antimicrobial metabolites, and demonstrated robust survivability throughout gastrointestinal transit. Consequently, it is inferred that these bacterial strains hold substantial promise as a biotechnological solution for *Salmonella* control in animal production.

**Abstract:**

Probiotics are increasingly recognized for their potential in managing bacterial challenges in animal production. This study aimed to evaluate the probiotic potential of *Bacillus* and *Streptomyces strains*, specifically their bioprotective ability against *Salmonella*. In agar inhibition assays, these bacteria supported *Salmonella*-inhibition zones, ranging from 2.5 ± 0.5 to 6.3 ± 2.0 mm. Analyses of antimicrobial metabolites revealed their capacity to produce compounds with anti-*Salmonella* properties, except for *Bacillus subtilis* MLB2. When *Salmonella* was exposed to lyophilized metabolites, inhibition occurred in both liquid (at concentrations between 250 and 500 g/L) and solid cultures (at 500 g/L). To confirm their probiotic potential, the *S. griseus* and *Bacillus* strains underwent evaluations for antimicrobial resistance, bile salt tolerance, auto- and co-aggregation, pH resistance, and their ability to adhere to and inhibit *Salmonella* in Caco-2 cells. These assessments confirmed their probiotic potential. The probiotic strains were further encapsulated and subjected to simulated swine and poultry digestion. They demonstrated survival potential through the gastrointestinal tract and significantly reduced the *Salmonella* population. Thus, these strains exhibit considerable promise for producing biotechnological products aimed at controlling *Salmonella* in animal production. This approach ensures the health and hygiene of farming facilities, mitigates the spread of zoonotic bacteria, and contributes positively to public health.

## 1. Introduction

One of the most significant issues in animal production are diseases caused by *Salmonella*. The transmission of *Salmonella* has been facilitated by the intensification of production and the increased density of animals within the same batch. The prevalence of *Salmonella* is particularly important in swine and poultry production. Research has indicated a prevalence of 26.3% in fecal samples collected from swine farms in China, one of the world’s largest producers [1]. In poultry production, data from farms in the United States, another major producer, indicate a prevalence of 46.2% [2].

Even in the absence of active disease manifestation, *Salmonella* remains a constituent of the commensal microbiome in production animals. Consequently, it is continually exposed to antibiotics, and is employed for therapeutic, prophylactic, or zootechnical purposes. This ultimately contributes to the high occurrence of antimicrobial resistance within this genus, posing a significant risk to public health. Presently, isolates exhibiting resistance to ampicillin, tetracycline, amoxicillin-clavulanate, trimethoprim-sulfamethoxazole, ceftriaxone, and ciprofloxacin have been identified [3,4]. The use of bioprotective microorganisms and/or probiotics are viable options for addressing this issue, managing the problem without creating long-term risks.

Probiotics are living microorganisms that, when administered in appropriate quantities, confer health benefits to the host. The main mechanisms of the action of probiotics include the restoration of intestinal microbiota, and competition for nutrients and space with potentially pathogenic bacteria, preventing their colonization and growth. Additionally, probiotics produce antimicrobial substances such as organic acids and bacteriocins, modulate the immune system by stimulating the production of cytokines and other immune mediators, strengthen the intestinal epithelial barrier, aid in digestion through the production of enzymes that facilitate the processing of complex molecules, and generate short-chain fatty acids linked to improved digestibility and nutrient absorption [5,6].

Probiotics prove to be highly beneficial as inhibitors of pathogenic bacteria. For example, Oliveira et al. [7] demonstrated the in vitro bioprotective potential of lactic acid bacteria against enterotoxigenic *Escherichia coli* isolated from swine production, Maes et al. [8] showcased the use of probiotic *Pseudomonas putida* against *Salmonella* Java biofilms for applications in poultry production, and Evangelista et al. [9] demonstrated the effectiveness of isolated lactic acid bacteria against *Salmonella* Typhimurium and *Salmonella* Enteritidis. This field is rapidly expanding, and various bacterial groups are associated with their potential use as probiotics [6]. In probiotic research, the Lactobacillaceae family receives considerable attention. Nevertheless, other bacterial strains exhibit noteworthy potential, and may serve as valuable assets in animal production, broadening the spectrum of available options. *Bacillus*, with its capacity for spore formation, holds significant promise for industrial processing. Additionally, bacteria belonging to the *Streptomyces* genus could represent important alternatives, given their prolific production of antimicrobial metabolites, further contributing to the expanding landscape of possibilities in this field.

In this context, the aim of this study was to assess the probiotic potential of different bacteria, characterize the antimicrobial metabolites produced in the culture medium, investigate the biocontrol exerted against *Salmonella*, and examine their survival in simulated swine and poultry digestion.

## 2. Materials and Methods

### 2.1. Strains and Materials

All culture media used were purchased from Oxoid (Hampshire, UK). All chemicals and reagents used were analytical or chromatographic grade, purchased from Fisher Scientific (Hudson, NH, USA) or Sigma-Aldrich (St. Louis, MO, USA). Ultra-pure water (~18.2 MΩ/cm resistivity) was obtained from a Milli-Q purification system (Merck Millipore, Darmstadt, Germany).

The potentially probiotic bacteria *Bacillus amyloliquefaciens plantarum* MLB3, *Bacillus subtilis* MLB2, *Bacillus velezensis* CL197, and *Streptomyces griseus* CECT 3276, and the pathogenic *Salmonella* strains *S.* Enteritidis CECT 23, *S.* Enteritidis CECT 160, *S.* Enteritidis CECT 161, *S.* Enteritidis CECT 162, *S.* Enteritidis CECT 163, and *S.* Enteritidis CECT 164, were used. The bacteria *B. amyloliquefaciens plantarum* MLB3 and *B. subtilis* MLB2 were obtained from the microbiological collection of the Biotech Agrifood Laboratory, University of Valencia, Spain. The bacterium *B. velezensis* CL197 was obtained from the microbiological collection of the Agri-Food Research and Innovation Laboratory, Pontifical Catholic University of Paraná, Brazil. The bacterium *S. griseus* CECT 3276 and the *Salmonella* strains were obtained from the Spanish Type Culture Collection, Spain.

The bacteria were preserved in tryptone soy broth (TSB) containing 20% glycerol at −20 °C until required. Prior to analysis, they were reactivated in the same culture media and incubated at 37 °C for 24 h, with a minimum of two passages.

### 2.2. Salmonella Inhibition in Culture Media

In vitro antimicrobial activity was assessed using an agar spot test [9]. Initially, the potentially probiotic bacteria were cultivated individually in TSB and incubated at 37 °C for 24 h. After bacterial growth, 3 µL of each culture was spotted onto the surface of tryptone soy agar (TSA) plates, followed by incubation at 37 °C for 24 h. Subsequently, aliquots of an overnight fresh culture of each *Salmonella* were inoculated into non-hardened TSA agar at 40 °C (population adjusted to ~10^7^ CFU/mL) and poured onto the TSA plates with the spots. The positive control consisted of *Salmonella* inoculated onto TSA without spots, while the negative control involved *Salmonella* inoculated onto TSA with spots of TSB only. The plates were subsequently incubated at 48 °C for 24 h, and the inhibition zones were measured. An inhibition zone equal to or greater than 1 mm in diameter was considered positive.

### 2.3. Antibacterial Metabolites’ Production and Characterization

Initially, the potentially probiotic bacteria were cultivated individually in TSB and incubated at 37 °C for 24 h. Then, the bacteria were inoculated in 200 mL of TSB (~10^4^ CFU/mL) and incubated at 37 °C in orbital shaker at 120 rpm. Aliquots were obtained after 0 h, 24 h, 48 h and 72 h, centrifuged at 3500× *g*, diluted with Milli-Q water (1:5 *v*/*v*), and filtered with nylon syringe filter with 0.22 µm pore size. To avoid interference in the growth process, different samples were prepared for each aliquoting time [10]. The samples were submitted to ultra-high performance liquid chromatography coupled with time-of-flight mass spectrometry (UHPLC-MS/qTOF) for antibacterial metabolites’ characterization.

Chromatographic analysis was performed on an Agilent 1200 HPLC (Agilent Technologies, Santa Clara, CA, USA), consisting of an auto sampler, vacuum degasser, and binary pump. Analyte separation was performed using a Gemini C18 column (50 mm × 2 mm, 110 Å, 3 μm particle size) (Phenomenex, Palo Alto, CA, USA). The mobile phases were water + 0.1% formic acid (solvent A) and acetonitrile + 0.1% formic acid (solvent B), with flow rate of 0.3 mL/min in gradient (0 min, 5% B; 30 min, 95% B; 35 min, 5% B), and a 25 min analysis run. The injection volume was 5 µL. For mass spectrometry analyses, a MS/qTOF (6540 Agilent Ultra High-Definition Accurate Mass, Agilent Technologies, Santa Clara, CA, USA), coupled with an Agilent Dual Jet Stream electrospray ionization (Dual AJS ESI, Agilent Technologies, Santa Clara, CA, USA) interface, operating in positive ion mode, was used. Optimized mass spectrometry parameters included: fragment voltage 175 V; capillary voltage 3.5 kV; collision energy 10, 20 and 40 eV, nebulizer pressure 30 psi; drying gas flow (N2) 8 L/min, and temperature of 350 °C. Data analysis was performed using MassHunter Qualitative Analysis Software B.08.00 (Agilent Technologies, Santa Clara, CA, USA) [11].

### 2.4. The Antimicrobial Activity of Metabolites

Initially, the potentially probiotic bacteria were cultivated individually in TSB and incubated at 37 °C for 24 h. Then, the bacteria were inoculated in 200 mL of TSB (~10^4^ CFU/mL) and incubated at 37 °C in orbital shaker at 120 rpm. Aliquots were obtained after 24 h, 48 h and 72 h, centrifuged at 3500× *g*, and lyophilized (Lab Freeze Drye, OLT-FD-10N, Xiamen Ollital Techoligy Co., Ltd., Xiamen, Fujian, China).

Initially, a bacterial growth inhibition analysis was performed on agar. For this purpose, 100 µL of a *Salmonella* inoculum containing 5 × 10^5^ CFU/mL was inoculated onto TSA plates. After complete drying, spots were made on the plates, with the addition of lyophilized metabolites, resuspended in ultrapure water at a concentration of 500 g/L. The plates were incubated at 37 °C for 24 h, and the zone of bacterial growth inhibition was measured. The negative control contained only ultrapure water, while the positive control consisted of the TSA plate with the bacterial inoculum, without treatments [12].

For the determination of antibacterial activity in liquid culture medium, the minimum inhibitory concentration (MIC) analysis was performed, with concentrations ranging from 0.98 to 500 g/L of lyophilized metabolites resuspended in TSB, using the microdilution technique. The wells contained 5 × 10^5^ CFU/mL of a *Salmonella* inoculum, with a final volume of 200 µL. The positive control contained only the bacterial inoculum, and the negative control contained sterile TSB. The plates were incubated at 37 °C for 24 h, and the lowest concentration with visible inhibition of bacterial growth was determined as MIC. After reading, the contents of wells with no visible growth were inoculated onto TSA plates. The lowest concentration required to kill the bacteria was defined as the minimum bactericidal concentration (MBC) [10].

### 2.5. Bacillus and S. griseus Probiotic Potential Confirmation

#### 2.5.1. Antimicrobial Resistance

Initially, the potentially probiotic bacteria were cultivated individually in TSB and incubated at 37 °C for 24 h. For the assessment of the antimicrobial resistance profile of the potentially probiotic bacteria, the technique of MIC by microdilution, as previously described, was used. The antibiotics vancomycin, gentamicin, kanamycin, streptomycin, erythromycin, clindamycin, tetracycline, and chloramphenicol were used for all bacteria, in addition to ampicillin for *S. griseus* CECT 3276. Interpretation was conducted according to the EFSA Panel on Additives and Products or Substances used in Animal Feed [13].

#### 2.5.2. Bile Salts Tolerance

Initially, the potentially probiotic bacteria were cultivated individually in TSB and incubated at 37 °C for 24 h. Then, the microorganisms (5 × 10^5^ CFU/mL) were individually exposed in 96-well plates to different concentrations (~0.04 to 20%) of bile extract in TSB. The plates were incubated for 24 h at 37 °C, followed by visual reading to determine the results. The highest concentration at which visual growth of the microorganisms occurred was determined as the maximum bile tolerance [14].

#### 2.5.3. Auto- and Co-Aggregation

Initially, the potentially probiotic bacteria were cultivated individually in TSB and incubated at 37 °C for 24 h. The fresh cultures of each microorganism were centrifuged (4000× *g*), and solutions with 10^9^ CFU/mL were prepared in sterile 0.9% saline solution. The samples were vigorously vortexed and incubated at 37 °C for 24 h without agitation. The absorbance at 600 nm of the solution was measured at 0 h, 3 h, 5 h, and 24 h. Auto-aggregation was determined according to the following formula, where AA (%) refers to the percentage of auto-aggregation, Abs_0_ to the initial absorbance (0 h), and Abs_t_ to the absorbance at 3 h, 5 h, or 24 h [7]:

$$AA\left(\%\right)=\left(\frac{{Abs}_{0}-{Abs}_{t}}{\begin{array}{c} {Abs}_{0} \end{array}}\right)\times 100$$

The co-aggregation analyses were carried out following the same protocol, with co-cultivation of each potentially probiotic microorganism and each *Salmonella* strain, and absorbance measurements at 0 h, 3 h, 5 h, and 24 h. Co-aggregation was calculated as follows, where CA (%) refers to the percentage of co-aggregation, Abs_0p_ to the initial absorbance of the potentially probiotic microorganisms, Abs_0e_ to the initial absorbance of *Salmonella*, and Abs_p+e_ to the absorbance of the co-culture at 3 h, 5 h, or 24 h [7]:

$$CA\left(\%\right)=\frac{\left[\frac{{Abs}_{0p}+{Abs}_{0e}}{2}-{Abs}_{p+e}\right]}{\frac{\left[{Abs}_{0p}+{Abs}_{0e}\right]}{\begin{array}{c} 2 \end{array}}}\times 100$$

#### 2.5.4. pH Resistance

Initially, the potentially probiotic bacteria were cultivated individually in TSB and incubated at 37 °C for 24 h. Then, the microorganisms (5 × 10^5^ CFU/mL) were individually exposed to TSB with pH adjusted to 2.0 ± 0.2, 3.0 ± 0.2, 8.0 ± 0.2, and 7.0 ± 0.2 (control) in 96-well plates. The samples were incubated at 37 °C for 24 h, and the growth was compared to the control using optical density at 600 nm [15].

#### 2.5.5. Adhesion to Intestinal Cell Lines and the Inhibition of Salmonella Adhesion

The inhibition of *Salmonella* adhesion when potentially probiotic microorganisms were used, and the adhesion of potentially probiotic bacteria to the intestinal epithelium, were evaluated according to Laparra and Sanz [16]. Caco-2 cells were inoculated into 96-well plates using DMEM High-Glucose medium + 20% fetal bovine serum, and incubated at 37 °C, 5% CO_2_ until they reached >90% confluence. To enable bacterial adhesion, cells were treated with mucin diluted in PBS according to the manufacturer’s recommendations, and 0.5 mL of this solution was added to the cell monolayer with incubation for 1 h at 37 °C, 5% CO_2_. After this period, excess mucin was removed by successive washes with PBS.

Three variations were performed: (i) potentially probiotic bacteria (used as controls in the *Salmonella* inhibition test and to evaluate the adhesion of potentially probiotic microorganisms to the cell monolayer); (ii) *Salmonella*; and (iii) co-culture. All groups contained a total volume of 200 µL of DMEM High-Glucose medium + 20% fetal bovine serum, with 10^6^ CFU/mL of the corresponding microorganisms.

The plates were incubated for 4 h at 37 °C, 5% CO_2_, and after the incubation period, cells were washed with PBS to remove non-adherent bacteria. Adherent bacteria were released by scraping, and their population was determined by inoculation onto TSA (for *Bacillus* and *S. griseus*) or MacConkey agar (for *Salmonella*) plates, and incubated at 37 °C for 24 h.

Based on the results obtained in the analyses described for the evaluation of probiotic potential, potentially probiotic microorganisms were treated as effectively probiotic microorganisms.

### 2.6. Probiotic Bacteria Encapsulation

In order to enhance the bacterial survival rate during gastrointestinal transit, the probiotic bacteria were encapsulated. The probiotic microorganisms were inoculated into TSB and incubated for 48 h at 37 °C. After incubation, the population was adjusted to 10^7^ CFU/mL, and 20% maltodextrin was added. Upon contact with the culture media, maltodextrin has the potential to form spherical structures, encapsulating the bacteria. The sample then underwent a non-lethal heat treatment (55 °C, 10 min), and was dehydrated by spray drying (flow rate of 12 mL/min and outlet air temperature of 70 ± 5 °C) (MSD/EV, Elettronica Veneta, Motta di Livenza, Italy). The resulting material had its microbial population determined to assess the effectiveness of the process through inoculation onto TSA [9].

### 2.7. The In Vitro Digestion of Encapsulated Probiotic Bacteria with Salmonella

#### 2.7.1. Bacterial Adaptation and Digestion Preparation

Before the in vitro digestion, *Salmonella* bacteria were acclimated to low water activity environments. They underwent multiple transfers in TSB enriched with 2% and 4% sodium chloride. These adapted bacteria were subsequently employed to create inoculants [17]. This acclimatization step was carried out to make the bacteria more representative of the conditions in animal production settings. Keeping isolates in nutrient-rich media can lead to a loss of virulence, pathogenicity, and adaptive traits. After adaptation, a 10^7^ CFU/mL inoculum was prepared in TSB, with all *Salmonella* combined. The population was standardized by optical density at 600 nm using a spectrophotometer.

Before the in vitro digestion, 50 g/L of encapsulated probiotic bacteria was individually reconstituted in sterile deionized water at 25 °C for 30 min, under magnetic stirring, until complete homogenization.

#### 2.7.2. Swine In Vitro Digestion

The in vitro digestion of swine was conducted in Falcon-type tubes at 39 °C and 200 rpm, mimicking the processes in the stomach and intestines. In the stomach phase, 1 mL of the reconstituted powder and 1 mL of the *Salmonella* inoculum were combined with 20 mL of phosphate buffer at pH 6.0, 8 mL of 0.8 M HCl, and 50 U of pepsin, and incubated for 2 h. After this incubation, the intestinal phase occurred in the same Falcon-type tubes with the addition of 7 mL of phosphate buffer at pH 6.8, 4 mL of 0.6 M NaOH, and 100 mg of pancreatin (4 × USP), followed by incubation for 4 h [18].

#### 2.7.3. Poultry In Vitro Digestion

The in vitro digestion of poultry was carried out in Falcon-type tubes at 42 °C and 200 rpm, mimicking the processes in the crop, proventriculus, gizzard, and small and large intestines. In the crop phase, 1 mL of the reconstituted powder and 1 mL of *Salmonella* inoculum were combined with 10 mL of sterile deionized water, adjusted to a final pH of 6.0 (using 1.0 M HCl). The tubes were then incubated for 30 min. In the proventriculus phase, the tubes were supplemented with 25 mL of sterile deionized water and 3850 U/mL of pepsin, and the final pH was adjusted to 2.5. The tubes were re-incubated for an additional 30 min. In the gizzard phase, glass beads were introduced to simulate mechanical digestion. The pH was adjusted to 3.0 with 1.0 M NaHCO_3_, and the tubes were further incubated for 1 h. For the small intestine phase, 8 U/mL of pancreatin and 135 mg of bile salts were added, and the pH was adjusted to 6.2. This phase required a 2 h incubation period. Finally, the large intestine phase was simulated by adjusting the pH to 7.0 and allowing for a 20 min incubation [19].

#### 2.7.4. Bacterial Recovery in In Vitro Digestion

*Salmonella* and probiotic populations were assessed at several points: before the stomach phase, after the stomach and intestinal phases in the swine in vitro digestion, before the crop phase, and after the gizzard and large intestine phases in poultry in vitro digestion. Enumeration was carried out by directly plating the samples (10^0^), and 10-fold serial dilutions on TSA and MacConkey agar. Plates were incubated at 37 °C for 24–72 h, and results were expressed as log CFU/mL. The positive control group was conducted without the addition of probiotics, while the negative control group was conducted without the addition of probiotics and *Salmonella*.

### 2.8. Statistical Analyses

Statistical analysis was performed using GraphPad Prism 8.0 (San Diego, CA, USA). Data were assessed for normality using the Shapiro–Wilk test, and exhibited a normal distribution; therefore, the results are presented as mean ± standard deviation. An analysis of variance by ANOVA was conducted, followed by the Tukey test. The significance level was set at *p* < 0.05.

## 3. Results

### 3.1. The Inhibition of Salmonella by Potentially Probiotic Bacteria in Agar Plates

All potentially probiotic bacteria effectively inhibited *Salmonella* on agar, with effects falling within the established methodological parameters (positive effect was indicated by the formation of a halo measuring equal to or greater than 1 mm). The inhibition generated by *B. amyloliquefaciens plantarum* MLB3 ranged from 2.5 ± 0.5 to 5.8 ± 1.7 mm, *B. subtilis* MLB2 exhibited inhibition from 3.8 ± 1.2 to 5.8 ± 2.1 mm, *B. velezensis* CL197 displayed inhibition ranging from 2.8 ± 0.7 to 6.3 ± 2.0 mm, and *S. griseus* CECT 3276 demonstrated inhibition from 2.8 ± 0.7 to 5.8 ± 1.9 mm. It is worth noting that in all analyses involving *Bacillus*, the *Salmonella* strain most affected was *S.* Enteritidis CECT 164, which might indicate an intrinsic susceptibility of this strain (Table 1).

### 3.2. Antibacterial Metabolite Production by Potentially Probiotic Bacteria, and Anti-Salmonella Effects

When evaluating the production of antibacterial metabolites by *Bacillus* in TSB, the production of several lipopeptides was detected; primarily, by *B. velezensis* CL197. Among the compounds produced by this strain, Macrolactin A and Bacillaene production stand out, with quantities of 7.12 ± 4.26 and 4.71 ± 0.28 mg/L, respectively, after 72 h of growth. No antibacterial metabolite production was detected for *B. subtilis* MLB2. It is worth noting that while some metabolites exhibit increasing production during the incubation period, such as Surfactin B produced by *B. amyloliquefaciens plantarum* MLB3, others have high production in the initial hours of growth, followed by degradation over time, such as Surfactin C produced by *B. velezensis* CL197. The same metabolite may present varying production and degradation profiles for different bacteria, as seen with Surfactin B, suggesting variable stability, possibly due to intrinsic differences among each strain, and their productive capabilities (Table 2).

The detection of metabolites produced by *S. griseus* CECT 3276 was carried out qualitatively, based on the metabolites listed in scientific databases about compounds produced by *Streptomyces*. However, this bacterium has extensive biotechnological applications and is used for the production of various compounds. Therefore, while this study demonstrated the high potential for metabolite production by the strain used, more comprehensive analyses are required for its complete metabolomic characterization (Table 3).

In the analyses of the antimicrobial activity of lyophilized metabolites in liquid culture media, the metabolites exhibited effectiveness mainly at doses of 250 and 500 g/L, with at least 48 h of bacterial growth. When using metabolites produced for 24 h, only those generated by *B. velezensis* CL197 and *S. griseus* CECT 3276 showed effects, which were of lower intensity, and were predominantly inhibitory. In the analysis performed with metabolites produced in 72 h of bacterial growth, all tests demonstrated inhibitory and bactericidal effects (Table 4).

In the agar inhibition analysis, the metabolites produced after 24 h and 48 h of bacterial growth were not effective in inhibiting *Salmonella*. However, with the metabolites produced after 72 h of bacterial growth, there was inhibition of *Salmonella* growth, with the formation of halos ranging from 2.6 ± 0.6 to 5.9 ± 0.5 mm. The minimum and maximum values refer, respectively, to the metabolites of *B. subtilis* MLB2 against *S.* Enteritidis CECT 162, and *B. velezensis* CL197 against *S.* Enteritidis CECT 162 (Table 5).

### 3.3. Probiotic Characterization

The analysis of antimicrobial susceptibility showed parameters within the guidelines established by the guidance on the assessment of bacterial susceptibility to antimicrobials of human and veterinary importance [13]. This demonstrates that, initially, these bacteria do not pose risks associated with antimicrobial resistance and the transmission of resistance genes. However, as there is considerable dynamism in the host’s organism following a potential future in vivo use, this susceptibility should be continuously evaluated to ensure that animals are not exposed to unknown risks (Table 6).

The assessment of bile salt tolerance also yielded favorable outcomes (Table 7). The existing literature has established that tolerance to concentrations of bile salts of at least 0.3% within the culture medium serves as a positive indicator of the bacteria’s ability to withstand the presence of bile salts in the host’s physiological conditions [39].

The analyses of auto- and co-aggregation are indicative of the potential of potentially probiotic bacteria to adhere to the intestinal epithelium, thereby creating a bioprotective film and inhibiting the adhesion of pathogenic bacteria. Additionally, they suggest the potential for the adsorption of pathogenic bacteria in the intestinal lumen, enabling their elimination through peristalsis. The bacteria used in the research exhibited high levels of both auto- (Table 8) and co-aggregation (Table 9). In the auto-aggregation results obtained after 24 h, there was variation from 36.56 ± 1.18% to 63.93 ± 6.81%, achieved by *B. velezensis* CL197 and *B. subtilis* MLB2, respectively. Meanwhile, co-aggregation analyses, after 24 h, yielded results of ~30% for all combinations performed.

Resistance to pH variations is one of the key characteristics associated with the survival of a probiotic in the gastrointestinal tract, especially given the extremely acidic pH encountered in the stomach and the sudden alkalization upon reaching the intestinal portion. Bacteria of the *Bacillus* genus exhibited approximately 50% growth relative to the control in an acidic pH, with proliferation exceeding the control when exposed to an alkaline pH. Although the viability of *Bacillus* decreased, its ability to sporulate may be an important factor in survival under different pH conditions. For *S. griseus* CECT 3276, there was no significant difference in bacterial growth, irrespective of the pH of the culture medium (Table 10).

The potentially probiotic bacteria exhibited a robust capacity for adherence to Caco-2 cells, with adherence percentages ranging from 10.1 ± 0.1% (adherence of *B. velezensis* CL197) to 11.3 ± 0.4% (adherence of *B. amyloliquefaciens plantarum* MLB3) concerning the initial bacterial population inoculated. The adherence of *Salmonella*, which in control groups ranged from 5.0 ± 0.7% to 7.6 ± 0.4%, was reduced to values between 2.2 ± 0.2% and 4.2 ± 0.4% when in competition with potentially probiotic bacteria (Table 11).

Based on the conducted analyses, the probiotic potential of the *Bacillus* and *S. griseus* strains has been confirmed.

### 3.4. Probiotic Encapsulation and In Vitro Digestion

The encapsulation methodology applied demonstrated its effectiveness by attaining an encapsulation efficiency ranging from 73.57 ± 9.43% to 86.71 ± 12.3%, concerning the original bacterial population within the liquid medium. The final bacterial populations were observed to range from 5.15 ± 0.66 to 6.07 ± 0.86 log CFU/g of the end product (Table 12).

The encapsulated probiotic bacteria demonstrated satisfactory survival rates during the simulated digestion processes. In the simulated swine digestion analysis, after 6 h of digestion, the recovered bacterial population in the intestinal phase ranged from 3.27 ± 0.31 to 4.77 ± 0.25 Log CFU/mL. In the simulated poultry digestion, at the end of the large intestine phase (4 h 20 min of digestion), the recovery varied from 4.47 ± 0.99 to 4.93 ± 0.36 Log CFU/mL. When competitive analyses were conducted between the probiotic bacteria and *Salmonella*, a significant reduction in the *Salmonella* population was observed in all analyses. In the swine digestion simulation, at the end of the intestinal phase, while the control group had a population of 6.65 ± 0.81 Log CFU/mL, in the treatment groups, it ranged from 2.69 ± 0.61 to 3.19 ± 0.67 Log CFU/mL. In the poultry digestion simulation, the control group displayed a population of 3.33 ± 0.23 Log CFU/mL, and in the treatment groups, it varied from 2.09 ± 0.48 to 2.45 ± 0.65 Log CFU/mL (Table 13).

## 4. Discussion

The bacteria used in this study, *B. amyloliquefaciens plantarum* MLB3, *B. subtilis* MLB2, *B. velezensis* CL197, and *S. griseus* CECT 3276, demonstrated satisfactory anti-*Salmonella* activity in all conducted analyses.

In the agar inhibition analysis with bacterial co-culture, inhibition zones ranging from 2.5 ± 0.5 to 6.3 ± 2.0 mm were observed. The results obtained in this study are consistent with findings published in previous studies. In vitro research has reported inhibition zones ranging from 3.3 ± 1.0 to 13.0 ± 1.4 mm when various *Bacillus* strains were used against *S.* Typhimurium [40]. Although similar studies with *S. griseus* against *Salmonella* were not found, scientific records suggest the antimicrobial efficacy of this bacterium [41,42].

When evaluating the production of antibacterial metabolites, no compounds were identified from *B. subtilis* MLB2. Previous articles available in scientific databases have shown a wide array of metabolite productions by *B. subtilis* strains [43]; therefore, the absence reported here may be a limitation of the employed technique. Although the applied technique allows for the presumptive detection of compounds, purified standards are necessary for effective confirmation, making the precise identification of molecules challenging. Although a wide range of metabolites, based on findings in the scientific literature, was sought, no antibacterial compounds were detected. More extensive analyses, particularly in the fields of metabolomics, proteomics, and genomics, should be conducted with this strain to possibly detect metabolites and address this knowledge gap. *B. amyloliquefaciens plantarum* MLB3 was capable of producing the metabolites bacillaene, macrolactin W, surfactin B, surfactin C, and 7,13-epoxyl-macrolactin A under the conditions used in this study, all of which have proven antimicrobial activity [44,45]. For *B. velezensis* CL197, the following metabolites were identified: bacillaene, macrolactin A, macrolactin F, surfactin B, surfactin C, surfactin D, and 7,13-epoxyl-macrolactin A.

Among the various metabolites produced, surfactins stand out as powerful biosurfactants with multiple biological activities. Among their antibacterial mechanisms, notable actions include attacking the pathogenic bacteria’s cell membrane, leading to cell membrane disintegration or osmotic pressure imbalance; inhibiting the protein synthesis of pathogenic bacteria, preventing cell reproduction; and inhibiting the enzyme activity of pathogenic bacteria, affecting normal cell metabolism [46].

Although there was a general trend of increasing production over the incubation period for most of the metabolites, surfactins B, C, and D exhibited a peak in production after 24 h of incubation, followed by degradation. Research has shown that surfactins are degraded throughout the production process, but when the bacteria enter the stationary phase, degradation becomes faster than production, leading to a decrease in the concentration of these compounds in the culture medium [47]. From the growth of *S. griseus* CECT 3276, a series of antibacterial compounds were presumptively detected. However, for the search of metabolites, a query of the available data in the scientific literature was conducted, and as bacteria of this genus are extensively used for the production of numerous compounds, both naturally and induced, it was not possible to conclusively establish the presence of the presumptively detected compounds. There is a possibility that these compounds are chemically similar, like isomers. Therefore, further in-depth research is needed to fill this knowledge gap.

The lyophilized metabolites demonstrated anti-*Salmonella* effects both in liquid and solid culture media. In liquid culture, when using metabolites produced during the first 24 h, only the metabolites from *B. velezensis* CL197 and *S. griseus* CECT 3276 displayed activity. There was a marked decrease in the metabolites without activity when applying metabolites produced over 48 h of incubation, and all metabolites were effective when produced over 72 h of incubation, with inhibitory and bactericidal doses of 250 and 500 g/L. In solid culture, there was no effectiveness of the metabolites produced during 24 and 48 h of incubation at the assessed dose (500 g/L). However, all metabolites produced during 72 h of incubation were effective, generating inhibition zones ranging from 2.6 ± 0.6 to 5.9 ± 0.5 mm. Once again, the results obtained here align with those found in scientific databases, which mention the antimicrobial activity of metabolites generated by *Bacillus* and *Streptomyces*, as previously mentioned. While the results have been positive, particularly with the metabolites produced during 72 h of incubation, it is worth noting the high doses required for *Salmonella* control. Although this information is scientifically relevant, high concentrations pose challenges for industrial applicability and the potential development of biotechnological products. With the confirmation of antimicrobial activity, genetic engineering techniques can be employed to enhance the production of desired metabolites. This may reduce the required dose for anti-*Salmonella* effects, thereby improving the industrial feasibility of these metabolites. Even though the use of metabolites in isolation is challenging, due to the high required doses, the research demonstrates that by providing these bacteria to animals, they have the potential to produce these compounds when they establish themselves on the intestinal epithelium.

The probiotic potential of these bacteria was satisfactorily confirmed, showing that the bacteria did not exhibit antimicrobial resistance, and could survive in the presence of bile salts and pH variations. They also demonstrated the potential to form bacterial aggregates, to adsorb Salmonella, and to create a bioprotective film on the intestinal epithelium, preventing the adherence of pathogenic bacteria. Research indicates that various *Bacillus* species have significant potential for use as probiotics, mainly due to their ability to form spores, which facilitates their handling and industrial survival, and survival during high-temperature processes. When employed as probiotics, *Bacillus* can offer not only antimicrobial effects but also anti-inflammatory, antioxidant, enzymatic, and immunomodulatory activities [48,49,50]. The *Streptomyces* genus has also demonstrated in previous research its potential for use as a probiotic, with proven antimicrobial, antitumor, antiparasitic, antifungal, and enzymatic effects [51,52]. However, a comprehensive characterization is essential for both bacterial groups to confirm their safety. There are *Bacillus* strains that can be pathogenic or toxigenic, and some *Streptomyces* produce yet-to-be-fully characterized metabolites. This underscores the need for ongoing research to ensure that animals are not exposed to unknown risks. Additionally, regulatory considerations for the bacteria evaluated in this study must be addressed. While *B. subtilis* and *B. amyloliquefaciens* already have regulatory approval for use as feed additives in animal production, *B. velezensis* is authorized only for agricultural use, and *S. griseus* lacks approvals for animal consumption or agricultural use. Comprehensive studies are, therefore, imperative to ensure the safe use of these bacteria and the development of biotechnological products.

The encapsulation technique employed in this study has proven effective in preserving bacterial viability. This method is easy and quick to apply, which can facilitate the development of biotechnological products based on the findings of this research. Previous studies conducted by our research group have shown that this technique maintains bacterial viability for a minimum of 12 months, with adequate encapsulation, ensuring that no bacteria remain on the external surface of the microcapsules [9]. Additionally, maltodextrin is a carbohydrate with prebiotic properties [53], which may induce beneficial effects through other pathways beyond those explored in this study. In simulated digestion, the probiotic bacteria demonstrated an adequate potential for survival through in vitro gastrointestinal transit, as expected based on previous tests. They also exhibited the anticipated anti-*Salmonella* effects, resulting in a significant reduction in the population of the pathogenic bacteria throughout the process. These effects were observed in both swine and poultry simulations, which allows for the future development of biotechnological products that can act similarly in both species.

## 5. Conclusions

The bacteria *B. amyloliquefaciens plantarum* MLB3, *B. subtilis* MLB2, *B. velezensis* CL197, and *S. griseus* CECT 3276 have demonstrated significant probiotic potential in vitro. They effectively inhibit the growth of *Salmonella*, resist the simulated gastrointestinal transit of swine and poultry, and exhibit adherence capabilities to the intestinal epithelial cell line Caco-2. Additionally, the metabolites Bacillaene, Macrolactin A, Macrolactin F, Macrolactin W, Surfactin B, Surfactin C, Surfactin D, and 7,13-epoxyl-macrolactin A were successfully detected in *B. amyloliquefaciens plantarum* MLB3 and *B. velezensis* CL197 cultures. Lyophilized culture media containing these metabolites demonstrated noteworthy anti-*Salmonella* activity. Metabolites of *B. subtilis* MLB2 were not detected, and only the presumptive detection of *S. griseus* CECT 3276 metabolites was possible. Despite incomplete characterization, lyophilized culture media with metabolites from both bacteria exhibited efficacy against *Salmonella*. This underscores the potential application of these bacteria, not only as probiotics but also as sources for postbiotic metabolites to *Salmonella* control within animal production.

To enable the application of *B. amyloliquefaciens plantarum* MLB3, *B. subtilis* MLB2, *B. velezensis* CL197, and *S. griseus* CECT 3276 in animal production, subsequent tests for in vitro characterization and in vivo research are essential. Among the necessary in vitro tests, it is imperative to assess its bacterial resistance to industrial handling processes, given that the manufacturing of animal feed typically involves high temperatures. Additionally, a comprehensive characterization of the metabolites produced by *B. subtilis* MLB2 and *S. griseus* CECT 3276 is required. Following the completion of in vitro tests, in vivo research become crucial, comparing the effectiveness of the studied bacteria with conventional methods of microbiological control. Thus, this research addresses fundamental aspects of probiotic investigation, providing new alternatives for enhancing animal production globally.

## Figures and Tables

**Table 1:** Inhibition of *Salmonella* Enteritidis (mm) on agar in the presence of spots of potentially probiotic bacteria.

Bacteria	*Bacillus amyloliquefaciens plantarum* MLB3	*Bacillus subtilis* MLB2	*Bacillus velezensis* CL197	*Streptomyces griseus* CECT 3276
*Salmonella* Enteritidis CECT 23	5.0 ± 2.7	4.2 ± 1.5	6.3 ± 2.0	5.2 ± 2.1
*Salmonella* Enteritidis CECT 160	5.2 ± 1.6	4.7 ± 2.6	4.3 ± 1.7	5.8 ± 1.9
*Salmonella* Enteritidis CECT 161	5.3 ± 1.4	4.0 ± 0.6	3.3 ± 0.8	2.8 ± 0.7
*Salmonella* Enteritidis CECT 162	5.8 ± 1.7	5.8 ± 2.1	5.5 ± 1.5	3.7 ± 1.9
*Salmonella* Enteritidis CECT 163	5.2 ± 0.7	4.7 ± 0.8	6.0 ± 0.6	5.2 ± 0.7
*Salmonella* Enteritidis CECT 164	2.5 ± 0.5	3.8 ± 1.2	2.8 ± 0.7	4.3 ± 0.8

**Table 2:** Production of antibacterial metabolites (mg/L) by potentially bioprotective *Bacillus*.

Incubation	Metabolites (mg/L *)							
	Bacillaene	Macrolactin A	Macrolactin F	Macrolactin W	Surfactin B	Surfactin C	Surfactin D	7,13-Epoxyl-macrolactin A
*Bacillus amyloliquefaciens plantarum* MLB3								
24 h	nd ^a^	nd ^a^	nd ^a^	nd ^a^	nd ^a^	nd ^a^	nd ^a^	0.38 ± 0.13 ^a^
48 h	0.61 ± 0.13 ^b^	nd ^a^	nd ^a^	nd ^a^	1.62 ± 0.26 ^b^	1.49 ± 0.41 ^b^	nd ^a^	1.01 ± 0.08 ^b^
72 h	0.57 ± 0.08 ^b^	nd ^a^	nd ^a^	0.25 ± 0.09 ^b^	2.76 ± 0.18 ^c^	1.72 ± 0.19 ^b^	nd ^a^	0.84 ± 0.17 ^b^
*Bacillus velezensis* CL197								
24 h	1.32 ± 0.20 ^a^	3.85 ± 2.34 ^a^	0.43 ± 0.12 ^a^	nd ^a^	5.57 ± 0.64 ^a^	11.92 ± 1.60 ^a^	1.53 ± 0.49 ^a^	nd ^a^
48 h	1.66 ± 0.19 ^a^	4.71 ± 2.77 ^a^	0.36 ± 0.07 ^a^	nd ^a^	3.35 ± 0.43 ^b^	7.55 ± 1.07 ^b^	1.02 ± 0.30 ^a^	nd ^a^
72 h	4.71 ± 0.28 ^b^	7.12 ± 4.26 ^a^	1.77 ± 0.06 ^b^	nd ^a^	1.25 ± 0.14 ^c^	3.20 ± 0.47 ^c^	nd ^b^	0.36 ± 0.07 ^b^

Different letters within the same column in the same group represent significant differences (*p* < 0.05) by the Tukey HSD test. nd: not detected. * Equivalent concentration in Surfactin C.

**Table 3:** Presumptive metabolites produced by *Streptomyces griseus* CECT 3276 detected in culture media after 24, 48, and 72 h of incubation.

Molecular Formula	Presumptive Compound	Ref.
C_14_H_25_N_3_O_9_	Kasugamycin	[20]
C_15_H_22_O	Albaflavenone	[21]
C_18_H_23_N_5_O_6_	Roseoflavin	[22]
C_18_H_34_N_2_O_6_S	Lincomycin	[23]
C_22_H_22_O_11_	Granaticinic acid	[24]
C_23_H_36_O_6_Na	Antibiotic E-975/AT37-1	[25]
C_24_H_27_NO_8_	Medermycin	[26]
C_24_H_30_O_6_	Peucemycin	[27]
C_26_H_33_ClO_5_	Merochlorin F	[28]
C_27_H_29_NO_6_	Frigocyclinone	[29]
C_3_H_7_O_4_P	Fosfomycin	[30]
C_32_H_24_O_9_	Bisanthraquinone BE-43472B	[31]
C_35_H_61_NO_12_	Oleandomycin	[32]
C_36_H_45_NO_12_	Chaxamycin D	[33]
C_36_H_53_N_3_O_10_	Lajollamycin	[34]
C_36_H_62_O_11_	Monensin	[35]
C_39_H_60_O_12_	Bafilomycin C1	[36]
C_39_H_65_NO_13_	Kitasamycin	[37]
C_5_H_5_NO_2_	1H-pyrrole-2-carboxylic acid	[38]

**Table 4:** Minimum inhibitory concentrations (MIC; g/L) and minimum bactericidal concentrations (MBC; g/L) of lyophilized culture media containing metabolites from potentially bioprotective bacteria, against *Salmonella*.

Bacteria	*Bacillus amyloliquefaciens plantarum* MLB3		*Bacillus subtilis* MLB2		*Bacillus velezensis* CL197		*Streptomyces griseus* CECT 3276	
	MIC	MBC	MIC	MBC	MIC	MBC	MIC	MBC
	24 h incubation							
*Salmonella* Enteritidis CECT 23	>500	>500	>500	>500	500	>500	500	>500
*Salmonella* Enteritidis CECT 160	>500	>500	>500	>500	500	>500	250	>500
*Salmonella* Enteritidis CECT 161	>500	>500	>500	>500	250	500	500	>500
*Salmonella* Enteritidis CECT 162	>500	>500	>500	>500	500	>500	250	>500
*Salmonella* Enteritidis CECT 163	>500	>500	>500	>500	500	>500	500	>500
*Salmonella* Enteritidis CECT 164	>500	>500	>500	>500	250	500	250	500
	48 h incubation							
*Salmonella* Enteritidis CECT 23	500	500	250	250	500	>500	500	500
*Salmonella* Enteritidis CECT 160	500	500	500	500	500	500	500	>500
*Salmonella* Enteritidis CECT 161	500	500	500	500	500	500	250	500
*Salmonella* Enteritidis CECT 162	500	500	250	500	500	500	250	500
*Salmonella* Enteritidis CECT 163	500	500	250	250	500	500	250	>500
*Salmonella* Enteritidis CECT 164	500	500	250	250	250	250	500	500
	72 h incubation							
*Salmonella* Enteritidis CECT 23	500	500	250	500	250	500	500	500
*Salmonella* Enteritidis CECT 160	250	500	500	500	500	500	500	500
*Salmonella* Enteritidis CECT 161	250	250	500	500	500	500	250	250
*Salmonella* Enteritidis CECT 162	500	500	250	500	250	500	500	500
*Salmonella* Enteritidis CECT 163	250	500	250	500	250	500	250	500
*Salmonella* Enteritidis CECT 164	250	500	250	250	500	500	500	500

**Table 5:** Inhibition of *Salmonella* Enteritidis (mm) on agar resulting from exposure to metabolites from potentially probiotic bacteria produced during 72 h of bacterial growth, lyophilized, and resuspended in ultrapure water (500 g/L).

Bacteria	*Bacillus amyloliquefaciens plantarum* MLB3	*Bacillus subtilis* MLB2	*Bacillus velezensis* CL197	*Streptomyces griseus* CECT 3276
*Salmonella* Enteritidis CECT 23	4.4 ± 0.5	4.9 ± 0.3	4.4 ± 0.6	4.3 ± 0.9
*Salmonella* Enteritidis CECT 160	3.0 ± 0.4	3.3 ± 0.7	4.1 ± 0.2	5.9 ± 0.4
*Salmonella* Enteritidis CECT 161	5.4 ± 0.4	5.1 ± 0.6	3.8 ± 0.9	5.0 ± 0.6
*Salmonella* Enteritidis CECT 162	4.6 ± 0.6	2.6 ± 0.6	5.9 ± 0.5	3.7 ± 0.3
*Salmonella* Enteritidis CECT 163	4.7 ± 0.9	2.8 ± 0.1	2.8 ± 0.5	5.2 ± 0.6
*Salmonella* Enteritidis CECT 164	3.3 ± 0.7	4.0 ± 0.2	4.0 ± 0.5	5.3 ± 0.7

**Table 6:** Antimicrobial susceptibility profile of potentially probiotic bacteria (mg/L).

Bacteria	AMP	VAN	GEN	KAN	STE	ERI	CLI	TET	CLO
*Bacillus amyloliquefaciens plantarum* MLB3	-	<0.062	0.125	0.500	1.000	<0.062	<0.062	2.000	0.250
*Bacillus subtilis* MLB2	-	1.000	4.000	8.000	4.000	0.250	0.500	<0.062	1.000
*Bacillus velezensis* CL197	-	0.250	0.250	4.000	4.000	<0.062	0.125	<0.062	4.000
*Streptomyces griseus* CECT 3276	1.000	2.000	4.000	0.250	8.000	0.500	0.250	0.500	2.000

AMP: ampicillin; VAN: vancomycin; GEN: gentamicin; KAN: kanamycin; STE: streptomycin; ERI: erythromycin; CLI: clindamycin; TET: tetracycline; CLO: chloramphenicol.

**Table 7:** Maximum concentration of bile salts (%) tolerated by potentially probiotic bacteria.

Bacteria	Maximum Tolerance (%)
*Bacillus amyloliquefaciens plantarum* MLB3	0.625
*Bacillus subtilis* MLB2	0.625
*Bacillus velezensis* CL197	1.250
*Streptomyces griseus* CECT 3276	0.625

**Table 8:** Auto-aggregation potential (%) of potentially probiotic bacteria.

Bacteria	3 h	5 h	24 h
*Bacillus amyloliquefaciens plantarum* MLB3	18.52 ± 4.84 ^a^	20.93 ± 4.29 ^a^	37.39 ± 0.16 ^b^
*Bacillus subtilis* MLB2	13.07 ± 5.68 ^a^	34.18 ± 2.00 ^b^	63.93 ± 6.81 ^c^
*Bacillus velezensis* CL197	18.34 ± 4.53 ^a^	20.44 ± 4.03 ^a^	36.56 ± 1.18 ^b^
*Streptomyces griseus* CECT 3276	12.76 ± 4.26 ^a^	13.25 ± 1.89 ^a^	45.05 ± 0.06 ^b^

Different lowercase letters in the same line denote significative difference (*p* < 0.05) by the Tukey HSD test.

**Table 9:** Co-aggregation potential (%) of potentially probiotic bacteria with *Salmonella*.

	*Bacillus velezensis* CL197					
	***Salmonella* Enteritidis CECT 23**	***Salmonella* Enteritidis CECT 160**	***Salmonella* Enteritidis CECT 161**	***Salmonella* Enteritidis CECT 162**	***Salmonella* Enteritidis CECT 163**	***Salmonella* Enteritidis CECT 164**
0 h	9.27 ± 0.11 ^a^	14.64 ± 0.72 ^a^	2.84 ± 0.37 ^a^	7.36 ± 1.10 ^a^	6.54 ± 0.71 ^a^	4.66 ± 1.80 ^a^
3 h	26.00 ± 0.12 ^b^	16.70 ± 0.15 ^ab^	15.41 ± 1.78 ^b^	31.53 ± 1.73 ^b^	6.61 ± 0.42 ^a^	7.61 ± 0.94 ^b^
5 h	32.97 ± 0.28 ^c^	17.80 ± 0.14 ^b^	25.74 ± 3.90 ^c^	31.76 ± 0.78 ^b^	9.48 ± 0.60 ^b^	7.92 ± 0.17 ^b^
24 h	33.76 ± 0.15 ^c^	33.84 ± 0.37 ^c^	34.18 ± 0.58 ^d^	32.52 ± 0.46 ^b^	33.63 ± 0.79 ^c^	29.40 ± 0.85 ^c^
	***Bacillus amyloliquefaciens plantarum* MLB3**					
	***Salmonella* Enteritidis CECT 23**	***Salmonella* Enteritidis CECT 160**	***Salmonella* Enteritidis CECT 161**	***Salmonella* Enteritidis CECT 162**	***Salmonella* Enteritidis CECT 163**	***Salmonella* Enteritidis CECT 164**
0 h	11.09 ± 2.96 ^a^	19.82 ± 0.96 ^a^	7.01 ± 1.21 ^a^	8.15 ± 1.76 ^a^	16.26 ± 3.11 ^a^	14.40 ± 4.12 ^a^
3 h	14.99 ± 2.17 ^a^	24.30 ± 1.18 ^b^	14.35 ± 3.77 ^b^	11.04 ± 1.82 ^a^	19.82 ± 3.01 ^a^	19.35 ± 3.17 ^a^
5 h	15.33 ± 2.11 ^a^	25.25 ± 1.26 ^b^	28.95 ± 4.65 ^c^	19.03 ± 3.32 ^b^	21.97 ± 2.80 ^a^	19.92 ± 0.04 ^a^
24h	29.64 ± 0.96^b^	29.21 ± 0.74 ^c^	33.46 ± 2.02 ^c^	25.99 ± 1.32 ^c^	36.17 ± 1.80 ^b^	29.27 ± 1.21 ^b^
	***Bacillus subtilis* MLB2**					
	***Salmonella* Enteritidis CECT 23**	***Salmonella* Enteritidis CECT 160**	***Salmonella* Enteritidis CECT 161**	***Salmonella* Enteritidis CECT 162**	***Salmonella* Enteritidis CECT 163**	***Salmonella* Enteritidis CECT 164**
0 h	5.68 ± 0.94 ^a^	15.55 ± 0.49 ^a^	8.15 ± 0.61 ^a^	3.55 ± 0.82 ^a^	6.09 ± 0.62 ^a^	5.30 ± 1.48 ^a^
3 h	20.16 ± 0.46 ^b^	20.57 ± 0.37 ^b^	10.67 ± 0.80 ^b^	18.72 ± 0.59 ^b^	6.16 ± 0.50 ^a^	6.70 ± 0.35 ^a^
5 h	33.07 ± 0.32 ^c^	29.49 ± 0.31 ^c^	29.50 ± 0.40 ^c^	31.24 ± 0.46 ^c^	18.63 ± 0.14 ^b^	14.72 ± 1.36 ^b^
24 h	36.12 ± 0.21 ^d^	32.09 ± 0.57 ^d^	30.83 ± 0.50 ^c^	33.80 ± 0.19 ^d^	26.17 ± 0.99 ^c^	25.57 ± 0.98 ^c^
	***Streptomyces griseus* CECT 3276**					
	***Salmonella* Enteritidis CECT 23**	***Salmonella* Enteritidis CECT 160**	***Salmonella* Enteritidis CECT 161**	***Salmonella* Enteritidis CECT 162**	***Salmonella* Enteritidis CECT 163**	***Salmonella* Enteritidis CECT 164**
0 h	2.07 ± 0.08 ^a^	4.45 ± 0.33 ^a^	3.84 ± 1.46 ^a^	3.07 ± 0.45 ^a^	4.85 ± 1.55 ^a^	2.79 ± 0.63 ^a^
3 h	20.38 ± 0.53 ^b^	25.00 ± 2.10 ^b^	20.67 ± 0.91 ^b^	21.30 ± 0.25 ^b^	23.76 ± 1.26 ^b^	22.07 ± 1.51 ^b^
5 h	29.70 ± 0.35 ^c^	34.23 ± 1.75 ^c^	28.27 ± 0.70 ^c^	29.04 ± 0.25 ^c^	33.20 ± 1.58 ^c^	30.46 ± 1.21 ^c^
24 h	36.34 ± 0.34 ^d^	34.99 ± 2.66 ^c^	34.17 ± 1.27 ^d^	34.73 ± 0.48 ^d^	36.01 ± 0.98 ^d^	33.70 ± 1.58 ^c^

Different lowercase letters in the same column, in the same group, denote significative difference (*p* < 0.05) by the Tukey HSD test.

**Table 10:** Bacterial growth (%) in different pH ranges, relative to the control (pH 7.0 ± 0.2).

Bacteria	pH 2.0 ± 0.2	pH 4.0 ± 0.2	pH 8.0 ± 0.2
*Bacillus amyloliquefaciens plantarum* MLB3	52.62 ± 6.58 ^a^	59.15 ± 3.29 ^a^	157.44 ± 30.48 ^b^
*Bacillus subtilis* MLB2	48.91 ± 6.40 ^a^	50.17 ± 2.31 ^a^	106.12 ± 11.62 ^b^
*Bacillus velezensis* CL197	57.08 ± 8.48 ^a^	48.69 ± 3.47 ^a^	134.16 ± 23.03 ^b^
*Streptomyces griseus* CECT 3276	106.33 ± 15.46 ^a^	106.33 ± 11.26 ^a^	97.24 ± 11.21 ^a^

Growth at pH 7.0 ± 0.2 = 100%. Different lowercase letters in the same line denote significative difference (*p* < 0.05) by the Tukey HSD test.

**Table 11:** Adherence (%) of potentially probiotic bacteria and *Salmonella* to Caco-2 cells, individually and in combination.

	Probiotic Control	*Salmonella* Enteritidis CECT 23	*Salmonella* Enteritidis CECT 160	*Salmonella* Enteritidis CECT 161	*Salmonella* Enteritidis CECT 162	*Salmonella* Enteritidis CECT 163	*Salmonella* Enteritidis CECT 164
*Salmonella* control	-	5.0 ± 0.7 ^a^	7.6 ± 0.4 ^a^	6.8 ± 0.9 ^a^	6.7 ± 0.4 ^a^	6.6 ± 0.8 ^a^	5.4 ± 0.4 ^a^
*Bacillus amyloliquefaciens plantarum* MLB3	11.3 ± 0.4 ^a^	3.7 ± 0.8 ^ab^	2.2 ± 0.2 ^b^	3.9 ± 0.7 ^b^	2.3 ± 0.9 ^b^	3.5 ± 0.2 ^b^	2.8 ± 0.7 ^b^
*Bacillus subtilis* MLB2	10.3 ± 0.5 ^a^	3.3 ± 0.2 ^b^	3.5 ± 0.3 ^c^	3.7 ± 0.7 ^bc^	2.3 ± 0.6 ^b^	2.9 ± 0.5 ^bc^	3.0 ± 0.2 ^b^
*Bacillus velezensis* CL197	10.1 ± 0.1 ^a^	3.5 ± 0.2 ^b^	2.8 ± 0.2 ^b^	2.8 ± 0.8 ^d^	2.6 ± 0.7 ^b^	2.8 ± 0.2 ^c^	2.4 ± 0.3 ^b^
*Streptomyces griseus* CECT 3276	10.2 ± 0.6 ^a^	3.8 ± 0.2 ^b^	3.7 ± 0.8 ^c^	3.1 ± 0.1 ^c^	2.4 ± 0.8 ^b^	3.6 ± 0.5 ^b^	4.2 ± 0.4 ^d^

Different lowercase letters in the same column denote significative difference (*p* < 0.05) by the Tukey HSD test.

**Table 12:** Encapsulation efficiency, analyzed immediately after the spray-drying process.

Bacteria	Log CFU/g	Efficiency (%)
*Bacillus amyloliquefaciens plantarum* MLB3	6.07 ± 0.86	86.71 ± 12.3
*Bacillus subtilis* MLB2	5.15 ± 0.66	73.57 ± 9.43
*Bacillus velezensis* CL197	5.81 ± 0.92	83.00 ± 13.1
*Streptomyces griseus* CECT 3276	5.78 ± 0.23	82.57 ± 3.28

**Table 13:** Survival of encapsulated probiotic bacteria in simulated swine and poultry digestion, and inhibition of *Salmonella* population when in combination (Log CFU/mL).

Bacteria	Swine In Vitro Digestion			Poultry In Vitro Digestion		
	0 h	2 h	6 h	0 h	2 h	4 h 20 min
*Bacillus amyloliquefaciens plantarum* MLB3	5.14 ± 0.81	4.46 ± 0.63	4.12 ± 0.07	4.50 ± 0.73	4.55 ± 0.63	4.93 ± 0.36
*Bacillus subtilis* MLB2	5.92 ± 0.80	5.29 ± 0.81	3.27 ± 0.31	3.49 ± 0.26	4.67 ± 0.72	4.47 ± 0.99
*Bacillus velezensis* CL197	5.29 ± 0.16	4.95 ± 0.78	4.77 ± 0.25	3.87 ± 0.60	4.73 ± 0.35	4.95 ± 0.41
*Streptomyces griseus* CECT 3276	5.93 ± 0.53	4.71 ± 0.49	4.38 ± 0.87	3.93 ± 0.22	4.33 ± 0.24	4.82 ± 0.03
*Salmonella*	7.28 ± 0.91 ^a^	7.76 ± 0.89 ^a^	6.65 ± 0.81 ^a^	6.75 ± 0.40 ^a^	5.43 ± 0.14 ^a^	3.33 ± 0.23 ^a^
*Bacillus amyloliquefaciens plantarum* MLB3 *+ Salmonella*	7.22 ± 0.28 ^a^	5.34 ± 0.70 ^b^	3.08 ± 0.17 ^b^	5.16 ± 0.62 ^b^	4.41 ± 0.59 ^b^	2.36 ± 0.17 ^b^
*Bacillus subtilis* MLB2 *+ Salmonella*	8.80 ± 0.89 ^b^	5.24 ± 0.60 ^b^	3.19 ± 0.67 ^b^	5.53 ± 0.54 ^b^	4.59 ± 0.71 ^b^	2.24 ± 0.20 ^b^
*Bacillus velezensis* CL197 *+ Salmonella*	7.40 ± 0.81 ^a^	4.27 ± 0.63 ^c^	2.97 ± 0.22 ^b^	6.90 ± 0.11 ^a^	4.17 ± 0.43 ^b^	2.45 ± 0.65 ^b^
*Streptomyces griseus* CECT 3276 *+ Salmonella*	7.62 ± 0.62 ^a^	4.98 ± 0.63 ^bc^	2.69 ± 0.61 ^b^	5.45 ± 0.93 ^b^	4.72 ± 0.39 ^b^	2.09 ± 0.48 ^b^

Different lowercase letters in the same column, in the same group, denote significative difference (*p* < 0.05) by the Tukey HSD test.

## Data Availability

All data supporting the findings of this study are available within the article.
